# The Role of Dietary Nutrients in Peripheral Nerve Regeneration

**DOI:** 10.3390/ijms22147417

**Published:** 2021-07-10

**Authors:** Marwa El Soury, Benedetta Elena Fornasari, Giacomo Carta, Federica Zen, Kirsten Haastert-Talini, Giulia Ronchi

**Affiliations:** 1 Department of Clinical and Biological Sciences, University of Torino, Orbassano, 10043 Torino, Italy; marwa.elsoury@unito.it (M.E.S.); benedettaelena.fornasari@unito.it (B.E.F.); giacomo.carta@gmail.com (G.C.); federica.zen@unito.it (F.Z.); 2 Neuroscience Institute Cavalieri Ottolenghi (NICO), Orbassano, 10043 Torino, Italy; 3 Institute of Neuroanatomy and Cell Biology, Hannover Medical School, 30625 Hannover, Germany; haastert-talini.kirsten@mh-hannover.de; 4 Center for Systems Neuroscience (ZSN), 30559 Hannover, Germany

**Keywords:** peripheral nervous system, nerve injury, micronutrients, macronutrients, dietary regimens, vitamins, minerals

## Abstract

Peripheral nerves are highly susceptible to injuries induced from everyday activities such as falling or work and sport accidents as well as more severe incidents such as car and motorcycle accidents. Many efforts have been made to improve nerve regeneration, but a satisfactory outcome is still unachieved, highlighting the need for easy to apply supportive strategies for stimulating nerve growth and functional recovery. Recent focus has been made on the effect of the consumed diet and its relation to healthy and well-functioning body systems. Normally, a balanced, healthy daily diet should provide our body with all the needed nutritional elements for maintaining correct function. The health of the central and peripheral nervous system is largely dependent on balanced nutrients supply. While already addressed in many reviews with different focus, we comprehensively review here the possible role of different nutrients in maintaining a healthy peripheral nervous system and their possible role in supporting the process of peripheral nerve regeneration. In fact, many dietary supplements have already demonstrated an important role in peripheral nerve development and regeneration; thus, a tailored dietary plan supplied to a patient following nerve injury could play a non-negotiable role in accelerating and promoting the process of nerve regeneration.

## 1. Introduction

Peripheral nerve injuries are very common, and it has been estimated that 3.3% and 1.8% of trauma patients with upper limb or lower limb injury respectively exhibit adjunctive nerve involvement [1,2]. Injuries to peripheral nerves affect over one million people every year worldwide [3] and may cause significant lifelong disability, with serious impacts (both physical and psychological) on the patients’ life. Associated annual healthcare costs for the treatment, care, and rehabilitation are very high, resulting in approximately €2.2 billion/year in Europe [4] and $150 billion/year in the United States [5].

Available strategies to improve axonal regeneration, neuronal survival, myelination, and reinnervation of target organs after nerve injury include surgical and non-surgical therapeutic approaches [6,7]. Depending on the type and severity of nerve injury, end-to-end repair, tubularisation techniques (autograft, allograft, and nerve conduit made from synthetic or natural materials), and nerve transfer are surgical repair strategies used in clinical practice [5]. Regarding non-surgical approaches, cell-based therapy performed with Schwann cells (SCs) or stem cells of different origin, electrical nerve stimulation, laser therapy, optogenetic therapy, and pharmacological therapy have been employed for promoting myelination and enhancing functional recovery after peripheral nerve injury [6,7,8,9,10]. Moreover, plant-derived compounds have been suggested to have potential beneficial effects on peripheral nerve regeneration [6].

Despite the progress in understanding the pathophysiology of peripheral nerve injuries, the advancement in reconstructive microsurgery, and the innovations in the fields of tissue engineering and regenerative medicine, there are no repair techniques or therapeutic approaches that can ensure full recovery of normal sensorimotor functions of adult patients following severe nerve injuries [11]. Therefore, alternative and potentially more efficacious treatments to ameliorate the regeneration of damaged peripheral nerves should be sought, but also supportive accompanying strategies are worth more consideration.

Dietary nutrients are essential for life and the maintenance of proper body functions; macronutrients (proteins, fats, and carbohydrates) provide the major sources of energy needed, while micronutrients (vitamins and minerals) play a central role in metabolism, e.g., through providing essential cofactors for enzymatic functions as well as the maintenance of specific tissue function.

Over recent years, scientific evidence has been obtained on the connection between dietary regimens or nutrients with the development and progression of different pathologies, including e.g., cardiovascular diseases [12], cancer [13], Alzheimer’s disease [14], Parkinson’s disease [15], and sarcopenia [16].

Recent studies have explored the role of single food components, as well as diets composed by different nutrients, in modulating the progression of nerve regeneration as recently reviewed by Yildiran et al. [17]). A better understanding of the role of specific nutrients could yield novel developments and breakthroughs in diet-based accompanying treatments for peripheral nerve injuries. Therefore, we aim here at critically reviewing the available knowledge about the role of nutrients in peripheral nerve regeneration.

## 2. Methods

**Figure 1:**
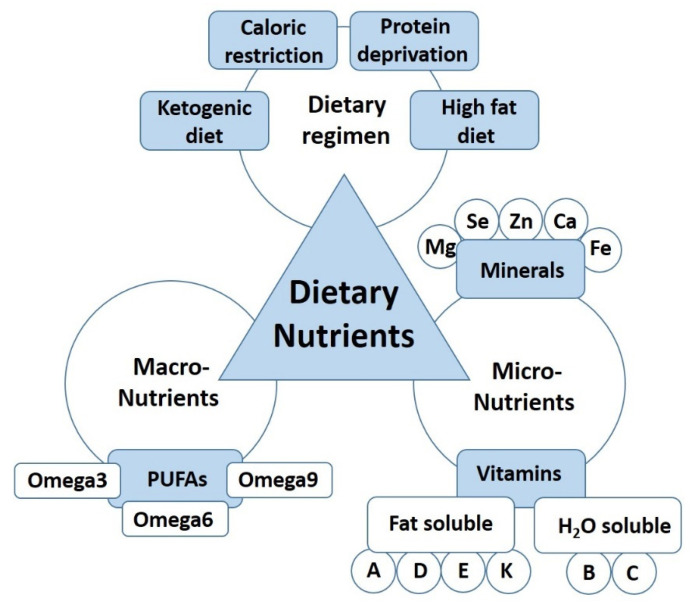
The dietary nutrients affecting nerve regeneration.

The literature was searched by using several e-sites, including PubMed, Embase, Scopus, Web of Science, and Google Scholar for work published until May 2021. Furthermore, bibliographies of all selected articles were screened in order to find any additional relevant paper. All selected articles were peer-reviewed and were published in English. We considered and discussed articles where the relationship between peripheral nerve regeneration and dietary regimens (such as ketogenic diet, high fat, or caloric restriction), macronutrients (protein, carbohydrates, and fats), as well as micronutrients (vitamins and minerals) was evaluated as summarised in Figure 1. Since we are aware that the studies that evaluate their hypotheses with the most comprehensive methods, including comprehensive analysis of nerve function, will have a considerably higher translational impact, we highlight those studies by presenting the results of the functional analysis in the main text.

We did our best to include all available articles; we apologise in advance if inadvertently we have missed some papers.

## 3. Dietary Regimen

The term ‘diet’ generally represents the sum of the consumed foods for maintaining body functions. Nowadays, it is clear that nutrition significantly affects the wellness and health of people and that it can assist in preventing or directly treating certain pathologies. Furthermore, it has been realised that the regeneration of the peripheral nervous system, one of the tissues most affected by damage during life, can also be influenced by nutrition. Although the currently available literature is still too different for providing a complete and systematic overview of dietary and nutritional effects, we present in the following paragraphs studies with a direct focus on dietary regimen and regeneration of the peripheral nervous system.

### 3.1. Ketogenic Diet

The ketogenic diet (KD) is an alimentary regimen that drastically reduces carbohydrates, while it increases fats and maintains an adequate minimum protein intake. The main purpose of this imbalance is to force the body systems to use fats as an energy source. Normally, ketoacidosis is a pathological state, but a nutritional induction of mild ketonemia for short periods seems to have positive effects in animal models, leading to improved metabolic profiles, extended lifespans, and improved neurological responses [18,19].

During KD, the liver produces the ketone bodies, which is a set of molecules serving as an alternative energy source to glucose that act in different ways on different processes [18,19]. It has been hypothesised that KD can affect the mitochondrial metabolism altering the genes involved in cellular respiration and decreasing the reactive oxygen species production [20].

Recently, the impact of KD on traumatic injuries to the spinal cord or peripheral nerves was reviewed by Sayadi et al. [21]. For completeness, given the purpose of this review, we report here the main results obtained with KD in the regeneration of peripheral nerves (summarised in Table 1).

Liskiewicz et al. [24] explored the impact of KD administered before and/or after sciatic nerve crush and observed that regenerating nerves in the preconditioned KD group showed the most similar morphometrical parameters to those of uninjured rats, including myelin thickness, fibre density, and fibre diameter. However, functional analysis based on the CatWalk test showed no significant differences between rats fed with KD and rats subjected to a standard diet.

More recently, Li et al. [22] evaluated the effects of KD in a rat sciatic nerve crush injury model with and without the electrical stimulation of the nerve. The results demonstrated the positive effect of KD on peripheral nerve regeneration and the positive synergistic effect with electrical stimulation. Indeed, all the analysed parameters (electromyography on biceps femoris muscle, mean myelin thickness, axon/fibre diameter, axon density, and walking corridor) were improved in rats receiving KD alone compared to controls and even more improved when KD was combined with electrical stimulation [22].

The effects of KD have also been examined with regard to sensorimotor recovery after complete transection of the common peroneal and tibial nerves in a mouse model [23]. The results displayed no significant differences between the dietary groups on a variety of motor function tests, including the open field test and ladder rung test. Moreover, the von Frey allodynia test showed no difference in recovery from mechanical allodynia in mice fed a ketogenic or standard diet. In this last study, the short duration of diet administration (7 days before and 28 days after surgery) maybe did not allow appreciating its effect on metabolism.

Overall, the lack of standardisation among all three studies in terms of diet composition, employed animal model, and timing of dietary intervention, as can also be derived from Table 1, precludes a synthesis of their outcomes as a whole. However, the coherent positive results reported by Liśkiewicz et al. and Li et al. [22,24] are noteworthy, because they performed their studies on the same animal model with similar timing and diet composition.

### 3.2. Caloric Restriction

Caloric restriction (CR) is a dietary reduction of energy intake. CR associated with adequate nutrition has been shown to extend health span and lifespan in rodent and primate models through metabolic and molecular adaptation and retardation of molecular damage accumulation [29]. In the last few years, the impact of CR on peripheral nerve structure and function with regard to age-related changes has been studied [30]. In particular, Rangaraju et al. [31] performed a study where SCs were obtained from young or aged nerves from rats kept on a standard diet or a CR diet (40% less daily consumption). The results indicated that a life-long CR regimen supports the maintenance of the molecular architecture of myelinated axons, including the expression of essential axonal and glial proteins, e.g., P0 and PMP22. More recently, Coccurello et al. and De Angelis et al. designed two different in vivo mouse studies for evaluating the effect of CR on peripheral nerve injury-induced neuropathic pain, with a specific focus on autophagy and aging, respectively [25,26]. In both studies, the mice were subjected to a procedure of monolateral chronic constriction injury (CCI) of the sciatic nerve and afterwards randomly assigned to standard diet or CR. The first study [26] showed that CR stimulated myelinogenesis through an acceleration of myelin debris clearance. Moreover, axonal growth protein GAP43 expression (indicative for regeneration processes) was strongly enhanced under CR in autophagy-defective heterozygous Ambra1 mice. The second study [25] used 12-month-old mice and clearly demonstrated a long-lasting decrease in hypersensitivity as induced by the peripheral nerve constriction lesion in rats under CR diet. An enhanced expression of GAP43 under CR was confirmed again. The morphological results emphasised that myelin proteins (P0 and PMP22) were evenly distributed and less aggregated under CR as compared with standard dietary regimen. However, a particular effect of CR on myelin protein expression was not described. The different results with regard to myelin gene expression can be explained by the different timing of administration of CR: the prolonged exposure to CR in [31] may have had a major effect on protein expression. Overall, caloric restriction seems to have a positive effect on nerve regeneration by acting in the early stages of regeneration.

### 3.3. Protein Deficient Diet (Protein Deprivation)

Although protein deficiency is one of the biggest public health problems in developing countries, there are no studies relating a protein-deficient diet to the regeneration of peripheral nerves after injury. On the other hand, a lot of studies between the 1960s and the 1980s have evaluated how malnutrition affects the peripheral nervous system in rats and humans. Oldfors [32] studied how protein deprivation in rats could affect the peripheral nerve morphology. The rats were subjected to severe protein deprivation from 6 to 27 weeks of age with a diet containing 1.5% protein, while the control group was fed with a diet containing 14% of protein. The protein-deprived rats showed markedly reduced serum albumin values and areas of alopecia. Nerve fibre material obtained from the tail displayed changes consistent with the Wallerian type of degeneration. More recently, Gomes et al. [33] investigated how a protein-restricted diet would affect the structure of the rat coeliac ganglion (CG). In rat fed with normal protein diet (20% casein), the CG consisted of larger neurons separated by non-neuronal tissue. Conversely, the CG neurons of undernourished rats (5% casein) were smaller and more densely distributed, demonstrating that a protein-deficient diet caused coeliac ganglion neurons atrophy and neuron loss. Coherently, Chopra et al. [34] observed a reduction of motor nerve conduction velocity and abnormalities of sensory conduction in children with different levels of protein malnutrition state. The abnormality of motor nerve conduction was directly related to the severity of malnutrition and the presence of hypotonia and/or hyporeflexia. Sural nerve biopsies were studied for myelinated fibre density, fibre size spectrum, and relationship of internodal length with diameter. In the biopsies from children with severe malnutrition, the normal developmental pattern for myelinated fibre size distribution was impaired with a persistence of small myelinated fibres, and there was a failure of internodal segments on large fibres to elongate with increase in age and significant demyelination.

The results of the reported studies Oldfors 1981 [32], Chopra et al., 1986 [34], and Gomes et al., 2009 [33] are consistent and demonstrated that a protein-deficient diet impairs peripheral nervous system development. So far, there are no studies available on the relation between protein deprivation and peripheral nerve regeneration. We can speculate that the peripheral nerve regeneration would be negatively affected, but further studies are needed for evaluating this hypothesis.

### 3.4. High Fat Diet

In developed countries, the percentage of obese people is increasing. Moreover, there is a change in diet composition due to the introduction of food with a high fat content. Many studies are demonstrating that a high-fat diet can become a risk factor for chronic diseases (i.e., prediabetes, obesity, hyperglycemia, peripheral neuropathy).

Regarding the effect of a fatty diet on rat sciatic nerve regeneration, no significant differences were detected in electrophysiological and functional tests (walking track analysis, hot plate test) [28]. However, morphological analysis showed that axon diameters and myelin sheath thicknesses of the standard diet injured group were significantly increased with higher amount of GAP43 immunopositive axons compared to the obese injured group [28].

A more recent study, performed by Song et al. [27], demonstrated that a high-fat diet increased postoperative pain and inflammation especially in males. They observed that a high-fat diet delayed skin wound healing and increased the density of regenerating nerve fibres near to the wound. Moreover, the intensity of GAP43 staining was four times higher in rats fed with a high-fat diet, and there was also delayed healing. A comprehensive analysis of the regenerated nerve is reported only by Bekar et al. [28]. The two studies are consistent in diet composition and, as a whole, indicative for a negative effect of a high-fat diet on peripheral nervous system regeneration and postoperative pain [27,28]. The findings with regard to GAP43 immunopositive axons were contradictory between Bekar et al. [28] and Song et al. [27]. Recently, although GAP43 is considered a biochemical marker for actively regenerating nerve fibres, it has been found to be associated with persisting neuropathic pain [35]. Therefore, it will be necessary to investigate the role of GAP43 in different peripheral nerve injury models and the timing of its expression.

## 4. Dietary Macronutrients in Peripheral Nerve Regeneration

### 4.1. Polyunsaturated Fatty Acids (PUFAs)

In the last four decades, evidence has been provided that in humans and mammals, the dietary supply of polyunsaturated fatty acids (PUFAs) has neuroprotective effects attenuating the neuronal damage after a traumatic or chemical nerve injury [36,37,38]. Under normal conditions, the endogenous eicosapentaenoic acid (EPA) and docosahexaenoic acid (DHA) total production is not enough to support all required physiological needs; for this reason, PUFAs must be included in the daily diet [39]. *ω*-3 PUFAs, which are enriched in foods such as avocado oil [40], olive oil, and pure fish oil [41,42], prevent neuronal death and reduce oxidative stress induced by reactive oxygen species after neuronal damage. Consumption of fish and fish products, which contain large amounts of DHA, significantly reduces the risk of ischemic events in the CNS and reduces the effects of neuronal ischemic damage when it occurs [42,43,44].

#### 4.1.1. Omega-3

It has been observed that ω-3 PUFAs are essentials for visual and neural development and that ω-3 dietary supply can induce neurological benefits when assessed in early or mild disease models [45]. The ω-3 fatty acids’ physiological functions in the nervous system are linked to the maintenance of cell membrane fluidity, which is fundamental for cell adhesion, dendritic formation, axonal guidance, synapse integrity, and synaptic conduction [46]. Several experiments on fat-1 mice, a transgenic mouse line overproducing ω-3 fatty acids, have shown that high levels of ω-3 PUFAs promote neuronal survival pathways and reduce oxidative stress and neuroinflammation [38,47,48]. Following low-dose radiation in rat fetuses, which is a model of systemic peripheral nerve degeneration, nerve damage and demyelination are prevented by the systemic administration of ω-3 PUFAs [49]. Considering pheochromocytoma cell lines (PC12) as a reliable model to study the autonomic nervous system cells’ behavior, the supply of DHA, C20:5n-3 EPA, and C20:4n-6 arachidonic acid can induce a significant increase in neurite growth [50].

Relevant evidence, suggesting a fundamental role in neuropathic pain modulation and nerve regeneration, is also provided by preclinical and clinical trials on human nerve injury paradigms. Indeed, as reported by Unda et al. (Table 2), the administration of a fish oil diet, enriched in ω-3 PUFAs, effectively relieves neuropathic pain and enhances the recovery process in rats with CCI of the sciatic nerve [51]. In particular, the regeneration index, which is defined as the total number of regenerated axons in the nerve segment distal to the ligation divided by the total number of regenerated axons in the nerve segment proximal to the ligation, was significantly higher in the group treated with enriched in ω-3 PUFAs diet than in non-treated animals. A double-blind randomised clinical trial on carpal tunnel patients has revealed that dietary supplementation of 600 mg/d of alpha-lipoic acid (ALA), an ω-3 PUFA, 1 month before and two months after surgery, significantly improved the neurophysiologic and clinical outcomes before and after surgery [52].

For ALA, an early antioxidant effect on the injured sciatic nerve has been demonstrated. In fact, while activities of the cellular antioxidants superoxide dismutase (SOD) and catalase (CAT) have been shown to be decreased significantly after crush injury compared to uninjured animals, intraperitoneal pretreatment with ALA, SOD, and CAT increased activities significantly, both at the first hour and on the 3rd day [56]. ALA has also a protective effect on peripheral nerve injury: oral administration of ALA after rat sciatic nerve injury led to higher sciatic functional index values compared to the untreated group, with increased axon and myelin size parameters, as well as fiber density at 30 days from the injury. Moreover, after ALA treatment, IL-1β and caspase-3 expression was downregulated, showing also the anti-inflammatory and antiapoptotic effects of ALA [55]. A more recent study confirmed the beneficial effect of post-injury treatment with ALA on nerve regeneration, showing faster functional recovery (better Sciatic Functional Index), improvement in connective and nerve tissue, and reduction in mast cell counts [53].

Therefore, ALA has been suggested to be an effective supplementary agent for the treatment of peripheral injuries.

DHA is a fundamental component of neuronal phospholipid membranes, and its deficiency is associated with impaired neuronal function and neurodegenerative diseases development [58]. DHA promoted axonal regrowth by enhancing in the nerve cells the gene expression of Bcl-2, Bcl-2-related protein A1 [48], and by attenuating caspase-3 downstream effects induced by oxidative stress [59,60]. DHA was also able to dampen inflammatory responses at the systemic level [61], and if administered intravenously, it had an analgesic effect in a neuropathic pain model of median nerve CCI, suppressing the c-Jun N-terminal kinase (JNK) signaling pathway [62]. DHA promoted neurite outgrowth and complex branching in rat dorsal root ganglia (DRG) dissociated cultures, independently from the age of the animals from which the cells were isolated [63]. As shown by Pham et al., DHA promotes early neurite outgrowth in the corneal nerve injury mouse model [64]. The DHA can also be combined with other PUFAs for promoting nerve regeneration. In particular, Silva et al. described how the daily oral administration of DHA/EPA-concentrate fish oil (Table 2) in mice with partial sciatic nerve ligation significantly reduced the neuronal damage in DRG, promoting a faster remyelination and functional recovery [54].

Taken together, all these studies support the beneficial effects of ω-3 PUFA in improving peripheral nerve regeneration after injury.

#### 4.1.2. Omega-6

ω-6 PUFAs, such as linoleic acid, are mainly converted by enzymes into ω-3 PUFAs [37] which, as previously described, are useful for cell adhesion, neurodevelopment, and neural plasticity [46]. The evening primrose oil, which is rich in the ω-6 essential fatty acid component, contains about 70% linoleic acid such as eicosanoid acid and 8–11% gamma-linolenic acid [57]. It has been demonstrated that esophageal feeding of evening primrose oil promoted nerve regeneration after sciatic nerve crush in rats, with significant faster recovery compared to untreated animal, which was demonstrated by a better toe-spreading reflex score. In addition, the morphological characteristics of axons and myelin were similar to those of healthy nerves at 4 weeks post nerve injury [57].

#### 4.1.3. Omega-9

Oleic acid (OA), which is rich in ω-9 PUFAs, is reported to be effective in preventing nerve demyelination in rat diabetic peripheral neuropathy induced by streptozotocin [65]. Indications for a supporting action of ω-9 PUFAs during peripheral nerve regeneration after trauma do not exist to our knowledge.

## 5. Role of Dietary Micronutrients in Peripheral Nerve Regeneration

### 5.1. Vitamins

Vitamins are a group of organic nutrients that are required in small quantities for a variety of biochemical functions that, generally, cannot be synthesised by the body and therefore must be supplied with the diet. Vitamins can be either classified into lipid-soluble or water-soluble vitamins. Lipid-soluble vitamins are hydrophobic compounds that can be absorbed efficiently just in case of normal fat absorption. They are transported in the blood within lipoproteins or attached to specific binding proteins. Lipid-soluble vitamins include vitamin A, D, E, and K.

The water-soluble vitamins are vitamins B and C; unlike the lipid-soluble vitamins, water-soluble vitamins are not stored in the body. Thus, they should be supplied daily. They function mainly as cofactors for some enzymes involved in the metabolism of carbohydrates, fats, and proteins [66].

#### 5.1.1. Lipid-Soluble Vitamins

##### Vitamin A and Retinoic Acid

Vitamin A (retinol) is a lipid-soluble essential vitamin that plays important roles in several biological cellular events, including differentiation, proliferation, survival, and death, and it plays also a role in maintaining tissue homeostasis [67]. It can be supplied to the human body in two different forms: as β-carotene taken from a vegetable source or as retinol esters taken from an animal source [67]. Then, both forms are converted to retinal or to retinoic acid (a carboxylic acid) through oxidation of the hydroxyl group [68,69].

Retinoic acid is the main metabolite exerting the function derived by Vitamin A intake through a series of oxidation reactions, all-trans retinoic acid (predominant isoform in most tissues) and 9-*cis*-retinoic acid are generated from it. All-trans retinoic acid acts by binding to the nuclear retinoic acid receptors (RAR), which heterodimerise with retinoid-X receptors (RXR) [70,71].

Many research articles suggest that vitamin A plays an important role during embryonic development, and its deficiency could even be lethal. A special role could be assigned to central nervous system development: the deficiency in vitamin A can cause severe deformation or even loss of the eyeball in newborn embryos [72]. Vitamin A is of great importance in molecular pathways underlying cognition and learning processes [73]. It was also found that vitamin A deficiency has a role in the development of psychiatric syndromes such as depression or schizophrenia and also in the development of Alzheimer disease [74].

The role of vitamin A in peripheral nervous system development or nerve regeneration in response to injury is less clear. As reviewed elsewhere, it was demonstrated that vitamin A is capable of supporting axonal outgrowth and neuronal survival likely mediated by the transcriptional activation of neurotrophin receptor genes [70].

In vitro studies with DRG (dorsal root ganglia)/SCs co-cultures and in vivo investigation of sciatic nerve development demonstrated that retinoic acid is a strong inhibitor of myelination in the peripheral nervous system, exerting its inhibitory action by two different mechanisms according to the activation of different retinoid receptors. The first acts on activating RXR that up-regulates Egr2 (Krox 20), which is a specific regulatory myelination transcription factor; the other acts by binding to RAR, resulting in the down-regulation of myelin-associated glycoprotein (MAG) [75,76].

Zhelyaznik et al. described that all the required components of the retinoic acid signaling pathway are expressed and activated in response to injury in adult rat sciatic nerves [77]. They also suggested a possible role of retinoic acid in regulating the neuregulin receptors ErbB2 and ErbB3 in response to nerve injury and thereby an indirect role of retinoic receptor regulation in SCs differentiation [78].

##### Vitamin D (Calciferol)

Vitamin D belongs to the fat-soluble vitamin family and is a steroid hormone precursor. It can be identified in two major forms: vitamin D2 (ergocalciferol) and vitamin D3 (cholecalciferol), which must be hydroxylated in liver and kidney for biological activation in tissues [79,80]. Vitamin D2 is produced in plants and fungi by their exposure to ultraviolet light, while most of vitamin D3 is produced after ultraviolet irradiation in most vertebrates [79,80]. The principal source of vitamin D is its endogenous synthesis in human skin through ultraviolet light exposure, but it can be provided through the diet in either their plant-derived (D2) or animal-derived form (D3) [81,82].

Vitamin D receptor is widely expressed in the human body [82]. Vitamin D is associated to a widespread number of biological processes, and its deficiency correlates with many different pathological conditions, which have recently been reviewed elsewhere [82,83,84,85,86,87,88,89,90].

Positive results with regard to peripheral nerve regeneration have been reported for the dietary intake effect of vitamin D (Table 3). The therapeutic potential of vitamin D2 was assessed in a rat model of 10 mm peroneal nerve transection repaired through the autograft technique [91]. The study demonstrated that ergocalciferol promotes axonal regeneration and increases axonal diameter. This improvement could be correlated to the regulatory effect of vitamin D on different neurotrophic factors involved in nerve regeneration [92,93,94], among which NGF demonstrated an increased synthesis. Moreover, in the vitamin D-treated group, a reduced degeneration of axons, proximal to the injury site, was observed, suggesting a neuroprotective role for vitamin D2; however, significant differences between vitamin D-treated and untreated animals in terms of functional recovery, as analysed by the peroneal functional index, were not detected [91].

Comparison of the effect of vitamin D2 and D3 on nerve regeneration demonstrated that vitamin D3 was more effective than vitamin D2 [95]. Indeed, peroneal functional index analysis demonstrated that cholecalciferol increased functional recovery more in comparison to ergocalciferol. This effect was dose dependent. Moreover, treatment with cholecalciferol resulted in increased diameter and number of axons and improved their myelination in comparison to ergocalciferol. Finally, microarray analysis on DRG and SCs stimulated for 24 h with calcitriol (biologically active form of vitamin D3) demonstrated that calcitriol up-regulated several myelin-associated genes in vitro [95].

**Table 3:** Lipid-soluble vitamins. Table showing the experimental method (type of nerve injury and animal model) and the dose and method of administration of the lipid soluble vitamins.

Reference	Type of Nerve Lesion and Animal Model	Type and Timing of Administration/Experimental Groups
**Vitamin D**		
Albay et al., 2020 [96]	Sciatic nerve crush (Wistar–Hannover female rats)	- B12 group: 1 mg/kg/day, intraperitoneal injection for 4 weeks - Vitamin D_3_ group: 3500 IU/week oral administration through gavage for 4 weeks - Vitamin B12 + D_3_ group: 1 mg/kg/day, intraperitoneal injection B12 and 3500 IU/week oral administration D_3_ for 4 weeks
Montava et al., 2015 [97]	Unilateral transected facial nerve repaired with autograft (New Zealand white rabbits)	From day 1 post-surgery weekly oral bolus of vitamin D_3_ (Uvedose^®^ 200 IU/kg/day Crinex). Treatment for 12 weeks.
Chabas et al., 2013 [95]	10 mm of the peroneal nerve cut out and repaired with autograft (Sprague–Dawley male adult rats)	Immediately after the injury and repair, rats were orally fed weekly (for eleven weeks) with boli containing: - Vitamin D2 at the dose of 100 IU/kg/day or 500 IU/kg/day - Vitamin D3 at the dose of 100 IU/kg/day or 500 IU/kg/day
Chabas et al., 2008 [91]	10 mm of the peroneal nerve cut out and repaired with autograft (Sprague–Dawley male adult rats)	3 times for month oral vitamin D_2_ administration (Sterogyl^®^ 100 IU/kg/day) for 12 weeks
**Vitamin E**		
Azizi et al., 2014 [98]	10 mm sciatic nerve transection unrepaired or repaired with chitosan conduit (Wistar male rats)	The chitosan conduit was filled with vitamin E (20 mg/kg, DL-all-rac-α-tocopherol) or pyrroloquinoline quinone (PPQ, 0.03 mmol/l) or a combination 1:1 of vitamin E and PPQ. Analysis were performed 4, 8, and 12 weeks after surgery.
Enrione et al., 1999 [99]	Sciatic nerve crush (Sprague–Dawley male rats)	Vitamin E-deficient diet: dl-α-tocopheryl acetate 0 mg/kg diet After 22 days of diet animals received nerve injury and continued feeding until sacrifice (15 days post-surgery).
Cecchini et al., 1994 [100]	Sciatic nerve crush (Sprague–Dawley male rats)	From day 30 after birth-deficient rats were fed a diet without vitamin E and injured at 3 months of age. Diet maintained until sacrifice (2 months from the injury).
Cuppini et al., 1993 [101]	Sciatic nerve crush (male Sprague–Dawley rats)	Vitamin E-deficient group: fed with a diet lacking vitamin E for 2 months before surgery. The dietetic treatment was maintained until the sacrifice (30 or 60 days after the lesion).
Cuppini et al., 1990 [102]	Sciatic nerve crush (Sprague–Dawley rats)	- Vitamin E-deficient group: fed with a diet lacking vitamin E - Control group: fed with a diet lacking vitamin E, supplemented with 120 mg/kg vitamin E After 2 months, some animals from all groups received nerve injury and continued feeding until sacrifice (30 or 60 days after the lesion)

The positive effect of vitamin D3 on functional recovery was also confirmed in rabbits after facial nerve transection and autograft repair. Weekly treatment with vitamin D3 reduced facial paralysis and improved myelination (in terms of *g* ratio) in comparison with vehicle treatment [97].

A recent study demonstrated improved values of the sciatic nerve functional index and reduced number of degenerating axons after sciatic nerve crush for either cholecalciferol (orally delivered) or cyanocobalamin (B12, water-soluble vitamin, see below, intraperitoneally injected) in comparison to untreated animals. Interestingly, combined therapy even resulted in a synergistic effect of further improved nerve regeneration [96].

##### Vitamin E

The vitamin E family is composed of eight different isoforms: α, β, γ, and δ tocopherols and α, β, γ, and δ tocotrienols, which share a chromanol ring. These natural vitamin E forms are plant-derived, and their principal dietary sources are vegetables and plant seeds, in particular vegetable oils and nuts [103,104].

All tocopherols and tocotrienols are potent lipophilic antioxidants protecting cell membranes with lipoperoxyl radical-scavenging activities [105,106,107,108]. Their prevention of free radical injury has been proposed as their major pathways for aging and diseases protection [108,109,110,111,112].

Different studies have been carried out to elucidate the effect of vitamin E on peripheral nerve regeneration (Table 3). Differences in muscular reinnervation after a sciatic nerve crush demonstrated that a higher number of motor endplates were polyinnervated, and this condition was maintained longer in rats on a vitamin E-deficient diet than in normally fed animals [101,102]. The enhanced terminal sprouting and the long-lasting polyinnervation of target muscles, which are conditions associated to nerve degeneration, could be attributed to a decreased protection from free radicals due to the vitamin E deficiency, which turn led to a delay in nerve regeneration.

The correlation between vitamin E deficiency and premature aging was strengthened further by the finding of an increased number of neurons in rat DRGs under vitamin E deficiency condition, which is an increase that is otherwise usually observed in older rats only [113,114], In a follow-up study, the vitamin E deficiency condition again increased the number of sciatic sensory neurons. However, differences in neuron loss after sciatic nerve crush injury were not observed between normally fed and rats on a vitamin E-deficient diet [100]. In contrast, Enrione et al. [99] demonstrated an impaired nerve regeneration, which was characterised by a reduced number of myelinated axons with thinner and irregular myelin in rats after sciatic nerve crush injury and under vitamin E-deficient diet in comparison to normally fed injured rats. An impaired myelination process could be attributed to myelin sheath oxidation associated to an increase in the oxidative stress following nerve injury.

More recently, the effect of vitamin E on peripheral nerve regeneration was also investigated after the repair of a 10 mm gap rat sciatic nerve injury repaired with a chitosan conduit [98]. The conduit was filled with vitamin E or pyrroloquinoline quinone (PQQ), which is a redox cofactor acting as antioxidant, or with a combination of the two molecules. Functional, morphological, and electrophysiological analysis demonstrated a significant improvement and acceleration in peripheral nerve regeneration that was even more pronounced in animals topically treated with vitamin E and PQQ combination. These findings confirmed that oxidative stress affects nerve regeneration and that it can be reduced by local vitamin E delivery [98].

##### Vitamin K

Vitamin K describes a family of fat-soluble compounds whose members have in common a 2-methyl-1,4-naphthoquinone ring. Its main members are vitamin K1 or phylloquinone (PK), vitamin K2 or menaquinones (MKn), and vitamin K3 or menadione, which differ in the side chains linked at the 3-position of the 2-methyl-1,4-naphthoquinone ring and in terms of function, target activity, and sources [115,116,117]. K vitamins are stored in a limited amount in the tissues, and their dietary intake should be supplied on a regular basis [118].

Vitamin K exerts relevant effects on different biological processes and physiological conditions [119], and its deficiency is linked to many pathological conditions, which are associated with inflammation and pathological mineralisation [115]. K vitamins are mainly used in clinical practice for blood coagulation and act as antioxidant and anti-inflammatory molecules, with a protective effect on CNS. In particular, a relevance for vitamin K has been postulated for the prevention of multiple sclerosis progression [120]. This is again related to the fact that oxidative stress has a crucial role in neurodegenerative diseases, and vitamin K seems to protect oligodendrocytes and neurons from oxidative injuries [121]. It further seems to be involved in remyelination after an acute demyelination [122,123].

Although the role of vitamin K in different biological processes is well known, few research articles documented its action on the peripheral nervous system. Indeed, rats after surgical removal (neurectomy) of the sciatic nerve were often used as a model for osteoporosis [124] and for investigating vitamin K effects on bone tissues but without taking into account its involvement in peripheral nerve regeneration. Tsang et al. [125] demonstrated that vitamins K1 and K2 enhance NGF-mediated neurite outgrowth of PC12D cells in vitro by activating PKA and MAPK-dependent signaling pathways. K vitamins (K1 or K2) alone did not induce PC12D neurite outgrowth, but when added together with NGF, the number of cells with neurite outgrowth was significantly increased in comparison to those treated with NGF only. To our knowledge, no further studies exist on vitamin K effects on the peripheral nervous system.

#### 5.1.2. Water-Soluble Vitamins

##### Vitamin B

Vitamin B is comprised of a group of water-soluble essential elements, which constitute eight different vitamins: B1 (thiamine), B2 (riboflavin), B3 (niacin), B5 (pantothenic acid), B6 (pyridoxine), B7 (biotin), B9 (folate), and B12 (cobalamin). Concerning their chemical composition, B vitamins are different, but they are considered as a group as they usually coexist and are supplied together from the same food [126,127]. Except for vitamin B3, which can be synthesised from tryptophan, all the other vitamins B are not synthesised by the human body; therefore, they must be supplied with the diet. Their direct supply derives from plants: mainly from rice, wheat, and other cereal crops. They can also be indirectly supplied through animal-derived food as meat, dairy, and eggs. Vitamin B12 is not produced by plants and can only be found in animal tissues or products such as meat, liver, fish, eggs, or dairy products, which have a wide variety of functions in the human body. Mainly the proper structure and function of the nervous system both central and peripheral depends directly on the mechanism of action of the vitamins B complex.

Members of the vitamin B complex mainly act as co-enzymes in various essential physiological processes. The members of the neurotropic B vitamin group (B1, B6, and B12) are well studied and well known to especially exert a positive role on the process of peripheral nerve regeneration (Table 4). Further specific studies will be needed for a better understanding of the exact role of the other members of the vitamin B complex as B2, B3, B5, and B7. Altun and Kurutaş showed that the vitamin B complex tissue levels, as detected by enzyme-linked immunosorbent assay, are altered in response to sciatic nerve crush injury [128]. The levels were significantly elevated at 1 and 12 h following nerve injury, while the level of vitamin B12 was significantly lowered at 1, 12 h, and 7 days compared to the control group [128]. Al-Saaeed et al. evaluated the rate of peripheral nerve regeneration in response to daily intramuscularly injection of different B vitamins (B1, B6, and B12). They found an increase in the number and density of regenerated myelinated fibers in the vitamin B-treated groups, in particular after treatment with vitamin B12, which was followed by vitamin B1 and vitamin B6 treatment [69,129]. A study by Ehmedah et al. demonstrated a modulatory effect on the macrophage–SC interaction during the early stages of Wallerian degeneration after nerve injury. Treatment with the B vitamin complex accelerated the transition from the non-myelin to myelin-forming SCs, which was accompanied by an increase in macrophage–SC interactions toward accelerating the transition from indispensable inflammation to nerve repair processes after peripheral nerve injury [130].

In the following, we report available knowledge on the different members of the vitamin B complex, always mainly focusing on peripheral nervous system studies. However, for many members of the vitamin B complex, specific reports in their effects on peripheral nerve regeneration do not exist so far.

**Vitamin B1 (Thiamine, aneurin)** acts as a co-enzyme in carbohydrates and branched-chain amino acids metabolism. It plays an important role in the three main energy pathways: pentose phosphate pathway, glycolysis, and Krebs cycle. Therefore, it largely contributes to the cellular energy metabolism providing energy to nervous tissue cells. These processes supply the nerves with energy mainly in the form of ATP or NADPH, which in turn are essential for numerous other cellular processes and reactions, both in neurons and glial cells [140,141]. Thiamine deficiency is the main cause of Beri Beri disease, which is a sensorimotor axonal neuropathy that is characterised by generalised weakness usually accompanied by sensory symptoms as paresthesias or limb numbness. Patients subjected to thiamine treatment either via oral consumption or injections have shown improvements in Beri Beri symptoms [142].

**Vitamin B2 (Riboflavin)** has co-enzyme functions in numerous oxidation and reduction reactions; it neutralises free radicals, such as reactive oxygen species, by maintaining glutathione in its reduced form and thus protects against harmful action of free radicals.

It supports the maintenance of a normal central and peripheral nervous system function and myelination. Moreover, it interacts with vitamin B6 and B9 and converts them into their active forms [143].

**Vitamin B3 (Niacin)** exists in two forms, nicotinamide and nicotinic acid; both act as a co-substrate/co-enzyme for hydrogen transfer with numerous dehydrogenases. It assists in carbohydrate conversion into glucose and aids in the production of fatty acids and cholesterol. It plays an important role in DNA replication and repair, as well as in cell differentiation; therefore, it is necessary for maintaining proper peripheral nervous system function [133,143].

**Vitamin B5 (Pantothenic acid)** is an immediate precursor to co-enzyme A and phosphopantetheine that is involved in fatty acid metabolism. Acetyl-CoA is necessary for the synthesis of the complex fatty-acyl chains of myelin; moreover, it also regulates iron levels, as proper iron levels are also essential for myelin production. Vitamin B5 deficiency is accompanied by peripheral nerve damage [133,143]

**Vitamin B6 (Pyridoxine, pyridoxamine, and pyridoxal)** functions mainly as a co-enzyme in the metabolism of amino acids, glycogen, and sphingolipids; in addition, the vitamin B6 complex possesses potent antioxidative characteristics as it effectively quenches reactive oxygen species [144,145]. Vitamin B6 deficiency is a causative factor in the idiopathic carpal tunnel syndrome, and its supplementation has been reported to alleviate the related symptoms as reviewed by Aufiero et al. [146].

**Vitamin B7 (Biotin)** functions as a co-enzyme in bicarbonate-dependent carboxylations, and it plays a key role in glucose metabolism. Thus, its deficiency mainly influences the brain, which is particularly sensitive to glucose metabolism and the delivery of glucose [133].

**Vitamin B9 (Folic acid)** improves peripheral nerve regeneration, as demonstrated by Hamra et al. and Sağır et al. Both studies showed improved electromyographic results and preserved axons with increased quantity and density and increased myelination after intraperitoneal injection of folic acid [131,134]. From an in vitro study by Kang et al., the improved regeneration may be attributable mainly to improved SC migration and proliferation as well as an increased secretion of NGF [132].

**Vitamin B12 (Cobalamin)** is a co-enzyme involved in many key metabolic pathways in lipid, carbohydrate, and protein metabolism. Cobalamin is usually consumed in the form of the co-enzyme de-oxadenosylcobalamin or methylcobalamin. Several studies have highlighted a positive effect of methylcobalamin and vitamin B12 on the myelination process by increasing the expression of myelin basic protein in SCs [147], thus accelerating the myelination process [69,129]. Furthermore, when administered together with dexamethasone (an anti-inflammatory glucocorticoid often prescribed after peripheral nerve injury) after traumatic sciatic nerve injury in rats, vitamin B12 increases the number of SCs and myelinated nerve fibers as well as the diameter of regenerating axons. This was accompanied by an increased sciatic functional index [136] or improved results in compound muscle action potential measurements, motor end plate counts, and axon and myelin analyses [135]. The proposed mechanism for the vitamin B12 action has been related to the increased expression of growth factors, such as brain-derived neurotrophic factor (BDNF) [136].

##### Vitamin C (Ascorbic Acid)

Ascorbic acid (Vitamin C) is a water-soluble vitamin with several important functions in the body, since it is necessary for the growth, development, and repair of all body tissues. Its functions include the synthesis of neurotransmitters and peptide hormones, regulation of transcription factors and gene expression formation of collagen, absorption of iron, the proper functioning of the immune system, wound healing, and the maintenance of cartilage, bones, and teeth.

Ascorbic acid, when systematically supplemented to the medium in axon–SC co-culture experiments, promotes in vitro myelination by enabling the SC to assemble a basal lamina, which is required for their differentiation [148] and by increasing DNA demethylation and the transcription of pro-myelinating genes [149].

Only recently, few studies focused on the effect of ascorbic acid administration on peripheral nerve regeneration following traumatic injury (Table 4). It has been demonstrated that oral administration of ascorbic acid improves the morphological and functional recovery of injured nerves [139]. Indeed, animals treated with ascorbic acid showed better motor and sensory performances as well as better electrophysiological results and larger myofibers in the target muscle after sciatic nerve crush injury. Moreover, supplementation with ascorbic acid increased the number and size of the regenerated axons and the myelin thickness. In vitro supplementation of ascorbic acid resulted in increased proliferation of SCs and upregulation of some neurotrophins [139]. Furthermore, the increased proliferation and migration capabilities of macrophages, which is another important player in nerve degeneration and regeneration, was reported [139]. The improvement in nerve regeneration after transection injury can be partially explained with a faster Wallerian degeneration observed in animals intragastrically treated with ascorbic acid: the fragmentation of axons and myelin is indeed promoted by ascorbic acid administration [138]. In turn, this can be explained by the effect of ascorbic acid on SC dedifferentiation (increased c-Jun-positive cells, a marker used to visualise immature SCs, and decreased levels of MAGs, a mature SC marker) on the one hand and on macrophages in the injured nerve on the other hand. Ascorbic acid promotes the clearing of axonal and myelin debris both in vivo and in vitro [138].

A possible mechanism of action of ascorbic acid in the peripheral nervous system has been hypothesised by using sodium-dependent vitamin C transporter 2-heterozygous (SVCT2+/−) mice. Deficiency of SVCT2 causes reduced ascorbic acid concentration in peripheral nerves, leading to hypomyelination and collagen-containing extracellular matrix deficits [150,151]. Sciatic nerve crush injury in these mice led to impaired functional performance and electrophysiological results with a defect/delay in remyelination; however, there were also normal regenerating axons and lower collagen I and collagen IV expression. In addition, the authors found an increased demethylation status of the collagen IV promoter region and increased Tet activity in ascorbic acid-treated DRG/SC cultures, and they therefore suggested that ascorbic acid may regulate collagen expression by Tet-dependent demethylation of collagen promoters [152].

Ascorbic acid is an essential nutrient in our diet, and it has been widely used as a dietary supplement and clinical drug [153,154]. The administration of ascorbic acid appears to be safe, convenient, and, based on recent findings, efficient in improving peripheral nerve regeneration. To date, no clinical trials are available on the administration of vitamin C in humans after traumatic nerve injury. So far, ascorbic acid supplementation has been tested in Charcot-Marie-Tooth 1A disease (CMT1A) patients with no significant effect [155].

### 5.2. Minerals

#### 5.2.1. Magnesium

Magnesium (Mg^2+^) is the fourth most abundant mineral, and it is essential for human health. Mg^2+^ plays important roles in the physiological function of basically every organ, including brain, heart, bone, blood, and skeletal muscle [156]. In addition, Mg^2+^ is the second most abundant intracellular cation and is involved in several metabolic and cellular processes in the body, including protein synthesis, cellular energy production and storage, reproduction, DNA and RNA synthesis and structure, and others. Moreover, Mg^2+^ acts as a physiological Ca^2+^ antagonist within cells: therefore, small changes in the Mg^2+^ availability may cause altered Ca^2+^ signaling or Ca^2+^ toxicity [157]. Mg^2+^ deficiency has been associated with a number of diseases, such as hypertension, migraine, depression, epilepsy, Parkinson’s disease, Alzheimer’s disease, cardiovascular disease, type 2 diabetes mellitus, preeclampsia, myocardial infarction, traumatic brain injury, and Mg^2+^ supplementation is considered as potential treatment for many of these conditions [157].

In recent years, researchers worked at identifying the effects of a low- or high-Mg diet on peripheral nerve regeneration (Table 5). It has been demonstrated that the administration of Mg^2+^ after axotomy improves nerve regeneration in terms of higher expression of neurofilament and S100 markers [158,159]. It further rescues sciatic motor neurons from axotomy-induced cell death [160,161] and muscle fibers from denervation-mediated atrophy, and it promotes motor-units survival [162]. Increased nerve regeneration in Mg^2+^ treated animals was also accompanied by an improvement in sciatic nerve functional index, increased compound muscle action potentials, and reduced nerve conduction latency [158,159].

It has been hypothesised that the beneficial effect of Mg^2+^ administration on nerve regeneration could be correlated with the inflammatory response. Indeed, Mg^2+^ depletion induces the release of inflammatory cytokines with a subsequent cascade of production of macrophage deposits that are detrimental to nerve regeneration. On the other hand, Mg^2+^ supplementation suppresses the inflammatory response and rescued SCs from apoptosis through an increase in the expression of Bcl-2 and Bcl-XL, which abolished the downstream expression of active caspase-3 and cytochrome C [158].

Another strategy that has been used to locally supply magnesium is the use of magnesium wire or filaments associated with a nerve conduit for repairing short (6 mm) as well as long-gap (15 mm) nerve injuries. Supportive effects were reported with regard to axonal and functional recovery [163,164,165]. However, further studies are needed to investigate the rate of degradation/resorption of Mg^2+^ implants in order to optimise the potential use of Mg^2+^ filaments in nerve repair approaches.

#### 5.2.2. Selenium

Selenium is a trace element, and it is an essential nutrient for human health. It is naturally present in many foods (especially grains and meat) and available as a dietary supplement. Selenium functions as a component of the antioxidant enzymes glutathione peroxidases, thioredoxin reductases, and iodothyronine deiodinases [170]; it is also a natural antioxidant [171] and has an important functional role in several body systems, including the central nervous system, male reproductive system, endocrine system, muscle function, cardiovascular system, and regulating immune cell functions [172].

In the last few years, due to its antioxidant property and neuroprotective effect (at low doses), researchers focused their attention on the effect of selenium administration on peripheral nerve regeneration (Table 5). It has been shown that selenium administration improved nerve regeneration after nerve compression in terms of electrophysiological and histological results (higher axon diameter, myelin thickness, and myelinated axon numbers) compared to untreated animals. The authors speculated that the preventive effects of selenium on axons and myelin could be mediated through blockage or reduction of the effects of lipid peroxidation chain reactions or through a positive effect on SCs due to a decrease in the effect of oxidative stress [166].

Two studies focused on the beneficial effect of intraperitoneal injection of selenium on damage caused by sciatic nerve ischemia–reperfusion [173,174]. The studies revealed that treatment with selenium increases the plasma activity levels of glutathione peroxidase and paraoxonase, decreases the plasma activity levels of nitrite [174], and decreases the inflammatory marker TNF-α in nerve tissue [173].

#### 5.2.3. Zinc

Zinc is an essential micronutrient for numerous metabolic processes, catalysing more than 100 enzymatic processes in the body. Current data suggest that zinc regulates multiple stages of neurogenesis, including cell proliferation, survival, and differentiation, and it is implicated in brain development, cognition, and regulation of mood [175].

Regarding the peripheral nervous system, it has been shown that sciatic nerves of animals fed with a zinc-deficient diet had smaller nerve cross-sectional areas, a decreased myelinated axon number [176], and decreased motor nerve conduction velocities [177] compared to animals fed with Zn-sufficient diet. In addition, abnormalities in nerve structure such as myelin and axonal damage were observed [176], as well as decreased Na, K-ATPase activity [177], showing that peripheral nerves require zinc for their development, survival, and function, but the mechanisms of action have not been elucidated yet.

No studies on peripheral nerve injury and regeneration have been carried out investigating the effect of local or systemic administration of zinc. However, some studies have explored the effect of Zn-containing devices both in vitro and in vivo, with the idea that these materials degrade and Zn ions (Zn^2+^) are released, with an effect on cells and nerve tissue [167,168,178,179]. Different approaches have been tested so far: (1) in vitro characterisation of an electrospun poly(ε-caprolactone) (PCL) matrix with different proportions of zero valent zinc nanoparticles [178]; (2) in vitro and in vitro analysis of Zn oxide-loaded polycaprolactone piezoelectric nanogenerator scaffold manufactured using 3D injectable multilayer biofabrication [167]; (3) in vitro evaluation of chitosan–zinc oxide nanocomposite conduit [168]; and (4) in vitro analysis of electrospun polycaprolactone with zinc chloride (ZnCl_2_) [179]. Beyond the approaches used, all these studies have shown a good biocompatibility and biodegradability of the devices, a positive effect on SC proliferation, and improved nerve regeneration, demonstrating that this type of approach could be suitable for supporting peripheral nerve regeneration.

#### 5.2.4. Calcium

Calcium is a major mineral nutrient in the human body, and more than 99% of it is stored in teeth and bone tissue, where it plays a key role in skeletal mineralisation. However, calcium plays also others numerous critical biological functions including the maintenance of healthy communication between the brain and other organs, muscle integrity and movement, and several cardiovascular functions [180].

Calcium has also been identified as an important signal that determines the advancement of growth cones and the rate of axonal outgrowth during neuronal development or regeneration [181].

In the field of peripheral nerve regeneration, different authors investigated the suitability of nerve conduits containing calcium in different forms to improve regeneration. In particular, the effect of calcium titanate (CaTiO_3_), a biocompatible ceramic with a perovskite structure, has been investigated in vitro. Nanoengineered porous chitosan/CaTiO_3_ hybrid scaffolds have been shown to be suitable for SC adhesion, proliferation, and biological function maintenance [182]. In addition, Zargar Kharazi et al. [183] designed a biodegradable nerve guidance conduit made of poly(glycerol sebacate) and CaTiO_3_ nanoparticles and evaluated the effect of calcium ion release on neurite outgrowth in vitro. They showed a non-toxic effect of calcium on PC12 cells and a good cell adhesion and proliferation with improved neurite outgrowth and extension. In vitro investigation of the suitability of the approach for improving peripheral nerve regeneration is outstanding.

Hydroxyapatite is a source of calcium with a slow-release pattern as well as excellent bioactivity and biocompatibility [169]. It has been used in association with both chitosan-based hydrogels [184] and with collagen type I hydrogels [169], and its good biocompatibility has been shown in vitro on murine fibroblasts L929 and human monocytic linage of THP1xBlue cells [184] and on SCs [169]. In addition, SCs showed higher proliferation compared to control, and the authors hypothesised that this was due to the release of calcium ions after nanoparticle degradation [169]. Finally, in vitro local administration of hydroxyapatite nanoparticle-containing collagen type I hydrogel on rat sciatic nerve crush injuries resulted in an enhanced functional recovery (sciatic functional index, hot plate latency, and compound muscle action potential) compared to negative controls; however, their complete function was not fully restored [169].

#### 5.2.5. Iron

Iron is an essential element for almost all living organisms, and it plays a crucial role in several functions, including oxygen transport, DNA synthesis and repair, electron transport, myelin formation maintenance, and for the synthesis of neurotransmitters [185,186]. Most of the iron in the body is employed in the transport proteins hemoglobin (in the blood) and myoglobin (in the muscular tissue).

Iron is also potentially toxic to cells since it can form free radicals. Therefore, it must be finely regulated, since excessive amounts can lead to accumulation with tissue damage [185].

An exhaustive review of the literature about the physiological role of iron in the peripheral nervous system and its involvement in peripheral neuropathy has been published a few years ago [185]. Here, the authors presented a detailed analysis of iron transport across the blood–nerve barrier, iron homeostasis in SCs, as well as its involvement in myelination. They also discussed the involvement of iron in disorders of the peripheral nervous system, including demyelinating and metabolic neuropathies.

More recently, the involvement of iron in myelin synthesis has been investigated further by using inducible conditional KO mice. Here, three proteins implicated in iron uptake and storage (the divalent metal transporter 1, DMT1, the ferritin heavy chain, Fth, and the transferrin receptor 1, Tfr1) were postnatally ablated, specifically in SCs. The results of these studies showed that the reduction in iron incorporation and storage reduces myelination both in vitro and in vitro [187].

Few studies have investigated the role and homeostasis of iron and iron uptake after peripheral nerve injury and during regeneration. In particular, it has been shown that transferrin is upregulated after nerve injury in SCs until 7 days from the injury [188] and in regenerating motor neurons [189], together with an elevated uptake of exogenous iron. In addition, intravenous injection of radioactive iron 59Fe^3+^ showed an increase in endoneural iron uptake in the lesion site and in the distal nerve, parallel to the local upregulation of transferrin receptor expression [190]. It has been proposed that transferrin could have a pro-differentiating role in SCs during nerve regeneration (and during late embryonic ages, where transferrin mRNA is also expressed in SCs) [188]. Furthermore, the enhanced capacity of regenerating motor neurons to bind transferrin and to uptake iron could play an important role in neural repair [189].

A little amount of data is available on the effect of iron deficiency diet on peripheral nerves. Iron deficiency anemia (IDA) is the leading cause for anemia, and it is the most common nutritional deficiency, affecting approximately two billion people globally [191]. The effects of IDA on peripheral motor nerve function has been investigated in human patients: this cross-sectional study revealed a functional impairment of peripheral nerves in condition of iron deficiency in terms of prolonged distal motor latency, reduced amplitude of compound muscle action potential, and reduced motor nerve conduction velocity [191]. Amos-Kroohs et al. [192] evaluated the effect of iron deficiency on rats’ peripheral nerves. They showed that iron deficiency caused a decrease in MBP and PMP22 expression, interfering with peripheral myelination.

All these data indicate a key role of iron in peripheral myelination during both development and remyelination after nerve injury. However, no data are available about the effect of iron deficiency diet or local or systemic administration of iron during nerve regeneration. These analyses could be very informative for further elucidating the role of iron and to develop new potential therapies based on iron to improve nerve regeneration.

## 6. Conclusions and Future Perspectives

A well-balanced diet is essential, as it provides the energy and nutrients required to survive and stay healthy. The combination of a healthy diet with a healthy lifestyle has huge benefits, and extensive evidence exists for their help in reducing the risk of many chronic diseases (e.g., heart disease, cancer, obesity, and diabetes).

**Figure 2:**
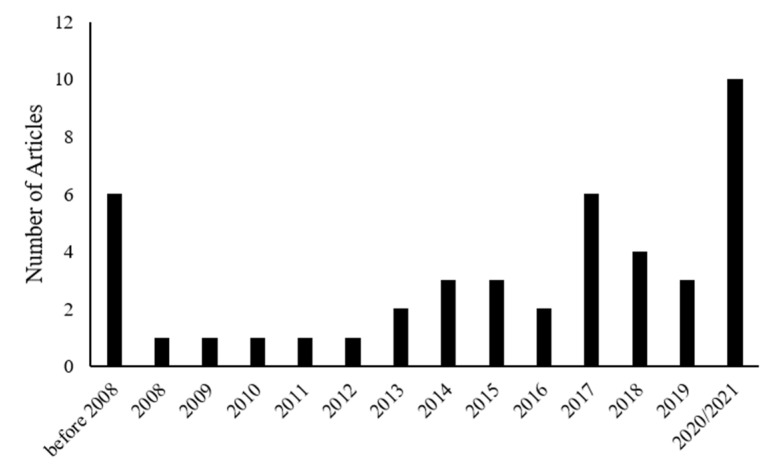
Graph showing the number of published papers about nutrition and peripheral nerve regeneration that were included in our manuscript divided by year of publication.

In recent years, there has been an increasing interest in investigating the effects of dietary nutrients on peripheral nerve regeneration. The growing number of articles published on the role of nutrients in nerve regeneration over recent years confirms the increasing attention in regeneration-promoting properties of the diet (Figure 2).

Many studies have shown the potential role of macro- and micronutrient supplementation in improving peripheral nerve regeneration. Interestingly, an even higher number of studies has detected the beneficial effects of micro- and macronutrient alimentation on the development or reduction of neuropathic pain, which is an event also related to peripheral nerve trauma. However, there are some points to be discussed before finally concluding on the benefit of dietary support for regenerative events in the peripheral nervous system.

First, most of the studies explored the role of a single food component in modulating the progression of nerve regeneration; however, people do not eat isolated nutrients but rather meals consisting of a variety of foods with complex combinations of nutrients that are likely to have synergistic effects. Of course, it is important to study the single nutrient in relation to nerve regeneration in order to identify its role in the process, but there are limitations, and further studies will be needed to elucidate the possible synergism between the different dietary factors. Few studies on different dietary regimens have been performed so far, and they highlight the beneficial effect of the ketogenic diet and caloric restriction diet, the detrimental effect of high-fat diet, and a hypothesised negative effect of the protein-deficient diet.

Second, several ways of nutrient administration following nerve injury have been used in the different studies (intraperitoneal injection/supplementation in food or water/oral gavage/local administration), with different doses and timing of administration. Therefore, it is difficult to suggest the best method of administration and the best dosage of the single nutrient because it is not possible, to date, to make comparisons between the different studies. The strategy of incorporating the nutrient in the medical device used to repair the injured nerve is very interesting, because it allows, through the degradation of the material, to have a controlled release of the factor over time in the site of nerve regeneration. This strategy has been used especially for minerals, but not for other nutrients, which is probably because the size and chemical nature of the minerals is more suitable for this type of approach. The different nutrients are essential for the proper functioning of the cells of the whole body, so a systemic administration would benefit not only the nervous tissue but also the whole body. However, it would be important to understand the amount of any nutrient factor that will reach the injury site following a systemic administration. In addition, the high-dose toxicity of some nutrients, such as iron, need to be taken in consideration for systemic administration.

Third, also the type of nerve injury differs among the different studies, ranging from mild crush injury to a more severe nerve injury with substance loss repaired with a conduit. The effect of the different nutrients on nerve regeneration could differ depending on the severity of nerve injury and timing of analysis after regeneration. In this regard, it will be of utmost importance to study the supplementation regime for the most promising micro- and macronutrient candidates in challenging preclinical models and comprehensive functional evaluation. From our literature review and its presentation above, the most promising candidates and administration routes are probably alimentation of the PUFAs ω-3 and ω-6, and alimentation of the vitamins D, B9, and B12, as well as the incorporation of the minerals Mg^2+^, zinc, and Ca^2+^ into scaffold materials for nerve repair.

Fourth, to have a better knowledge and to improve the potential therapeutical effects of dietary nutrients, the mechanism of actions on a molecular level and the activated molecular pathways need to be investigated in more detail. It seems that most of the nutrients act by reducing free radicals and activating Schwann cell–macrophages cross talk, but more studies are necessary to elucidate the molecular mechanisms and underlying cellular responses.

In conclusion, dietary nutrients cannot substitute conventional treatments, but a healthy diet with an appropriate intake of micro- and macronutrients may ameliorate the rate and degree of peripheral nerve regeneration after trauma and surgical repair. The findings that several nutrients improve nerve regeneration (both from a morphological and functional point of view) suggest that an appropriate nutritive intervention may worth being considered more as a possible complementary strategy in the treatment of nerve injuries in the future.

## Figures and Tables

**Table 1:** Dietary regimens. Table showing the experimental method (type of nerve injury and animal model) and the different dietary regimens.

Reference	Type of Nerve Lesion and Animal Model	Description and Timing of the Diet
**Ketogenic Diet**		
Li et al., 2020 [22]	Sciatic nerve crush (Sprague–Dawley rats)	65.8% fat, 3.0% carbohydrate, and 18.1% protein for 8 weeks after injury
Mayr et al., 2020 [23]	Transection of the common peroneal and tibial nerves in mouse (C57/BL6 mice)	75.1% fat, 3.2% carbohydrates, and 8.6% protein for 7 days before injury and up to 28 day after injury
Liśkiewicz et al., 2016 [24]	Sciatic nerve crush (Wistar rats)	79% fat, 0.8% carbohydrates, and 9.5% protein for 6 weeks after injury. Preconditioned group received KD also for 3 weeks before injury
**Caloric restriction**		
De Angelis et al., 2020 [25]	Chronic constriction injury of sciatic nerve in mouse (CD1 male mice 12 months old)	40% less of daily consumption for 7 days after CCI
Coccurello et al., 2018 [26]	Chronic constriction injury (CCI) of sciatic nerve (Wild-type CD1 male mice, Ambra1+ transgenic CD1 male mice)	40% less of daily consumption for 7 days after CCI
**High-fat diet**		
Song et al., 2018 [27]	Plantar incision (Long-Evans rats)	40% fat administrated with different timing: a group received a high-fat diet for 6 weeks before injury and 2 weeks after, another group was fed with high-fat diet for 7 weeks and then switched back to standard diet for 2 weeks before to plantar incision, while in the last group, only male rats were switched to high-fat diet 1 week before injury
Bekar et al., 2014 [28]	Sciatic nerve crush injury (Sprague–Dawley rats)	40% fat for 3 months (started before injury and ended 4 weeks after injury)

**Table 2:** PUFAS. Table showing the experimental method (type of nerve injury and animal model) and the dose and method of administration of the PUFAS.

Reference	Type of Nerve Lesion and Animal Model	Type and Timing of Administration/Experimental Groups
**Omega-3**		
Oktay et al., 2020 [53]	Sciatic nerve crush (Sprague–Dawley male rat)	50 or 100 mg/kg intraperitoneal alpha-lipoic acid (ALA) injection was done daily after nerve injury for 14 days
Unda et al., 2020 [51]	Sciatic nerve chronic constriction injury (Wistar rats)	Saline solution with concentrated salmon oil *ω*-3 (0.72 g/kg) administered orally for 21 consecutive days
Silva et al., 2017 [54]	Partial sciatic nerve ligation model (Swiss mice)	DHA/EPA-concentrate fish oil 2.3 g/kg orally administered 5 days/week for 2 weeks after nerve injury
Demir et al., 2014 [55]	Sciatic nerve crush (adult female albino Wistar rats)	ALA was suspended in distilled water 1 mL (per rat and administered orally at a dosage of 25 or 50 mg/kg by gavage), daily for 30 days
Senoglu et al., 2009 [56]	Sciatic nerve crush (female Sprague–Dawley rats)	100 mg/kg intraperitoneal ALA injection was done 24 and 1 h before crush injury
**Omega-6**		
Ramli et al., 2017 [57]	Sciatic nerve crush injury (Sprague–Dawley rats)	6000 mg/day of evening primrose oil through an esophageal feeding tube starting from day 1 after nerve crush for 4 weeks

**Table 4:** Water soluble vitamins. Table showing the experimental method (type of nerve injury and animal model) and the dose and method of administration of the water-soluble vitamins.

Reference	Type of Nerve Lesion and Animal Model	Type and Timing of Administration/Experimental Groups
**Vitamin B complex**		
Ehmedah et al., 2020 [130]	Transection of the motor branch of femoral nerve followed by end-to-end repair (Albino Oxford adult male rats)	Intraperitoneal injection of vitamin B complex ampoules (2 mL) of Beviplex (Beviplex^®^, Galenika a.d. Belgrade, Serbia), each containing B1 (40 mg), B2 (4 mg), B3 (100 mg), B5 (10 mg), B6 (8 mg), and B12 (4 µg). Dose: 1.85 mL/kg/day. The first was injected immediately (15 min) after the surgery and then every 24 h from the day of the operation until the day of sacrifice. Sacrifice: 1, 3, 7, and 14 days after surgery.
Sağır et al., 2020 [131]	Sciatic nerve crush (Wistar male rats)	Daily folic acid intraperitoneal injection for 6 weeks (0.5 mg/kg/day)
Al-Saaeed et al., 2019 [129]	Sciatic nerve crush (Albino male rats)	- Vitamin B1intramuscular injection of 180 mg/kg - Vitamin B6 intramuscular injection of 180 mg/kg - Vitamin B12 intramuscular injection of 1 mg/kg - Co-administrated injection of Tri-B (B1, B6, and B12), 20 mg/kg/day Daily treatment for 15, 30, and 45 days
Kang et al., 2019 [132]	End-to-end repair of the sciatic nerve (Sprague–Dawley male rats)	Intraperitoneal administration of 80 μg/kg folic acid for 7 days following nerve injury
Nedeljković et al., 2018 [133]	Transection of the motor branch of the femoral nerve with immediate reconstruction using a termino-terminal anastomosis (Albino Oxford rats)	Treatment with vitamin B complex (Beviplex^®^, Galenika a.d. Belgrade, Serbia). Each ampoule (2 mL) contains B1 (40 mg), B2 (4 mg), B3 (100 mg), B5 (10 mg), B6 (8 mg), and B12 (4 µg). The dose was 1.85 mL/kg/day. Administration by intraperitoneal injection daily from the day of injury until the day of sacrifice. Examination at 1, 3, 7, and 14 days after injury.
Al-Saaeed et al., 2015 [69]	Sciatic nerve crush (Albino rats)	- Vitamin B1 intramuscular injection of 180 mg/kg - Vitamin B6 intramuscular injection of 180 mg/kg - Vitamin B12 intramuscular injection of 1 mg/kg Daily treatment for 45 days
Harma et al., 2015 [134]	Tibial nerves transection repaired by end-to-end repair (adult male Wistar Albino rats)	Intraperitoneal administration of 80 μg/kg folic acid daily for 6 weeks
Liao et al., 2013 [135]	End-to-side neurorrhaphy between ulnar and musculocutaneous nerves (Wistar male rats)	Methylcobalamin (500 µg/mL; Methylcobal^®^ Eisai, Tokyo, Japan) 250 μg/day Daily intraperitoneal injection for 3 months
Sun et al., 2012 [136]	Sciatic nerve crush of the peroneal branch (Wistar male rats)	Injection into the injured site of vitamin B12 alone (2 mg/kg) or with dexamethasone (1 mg/kg), daily for 28 days
Okada et al., 2010 [137]	Sciatic nerve cut followed by direct end-to-end suture (Wistar female rats)	Continuous administration of methylcobalamin (1 mg/kg/day) in PBS for 12 weeks using an osmotic minipump placed subcutaneously in the right side of the back
**Vitamin C**		
Li et al., 2021 [138]	Sciatic nerve transection (Specific-pathogen-free female Sprague–Dawley rats)	Intragastric administration of ascorbic acid solution (suspension with saline at a concentration of 20 mg/mL): 400 mg/kg the day of injury; 200 mg/kg the following days for 5 or 8 days
Li et al., 2019 [139]	Sciatic nerve crush (C57BL/6 mice)	Daily intragastric administration of ascorbic acid solution (suspension with saline at a concentration of 13.33 mg/mL): 400 mg/kg the day of injury; 200 mg/kg the following days for 3 or 28 days

**Table 5:** Minerals. Table showing the experimental method (type of nerve injury and animal model) and the dose and method of administration of the minerals.

Reference	Type of Nerve Lesion and Animal Model	Type of Administration/Experimental Groups
**Magnesium**		
Cheng et al., 2019 [159]	Sciatic nerve crush (Sprague–Dawley rats)	High magnesium water (magnesium salt solution (1 mg/mL) after nerve injury) for 4 weeks
Hopkins et al., 2017 [163]	6 mm sciatic nerve defect repaired with 8 mm long poly(caprolactone) (PCL) nerve conduit AND 15 mm sciatic nerve defect repaired with 17 mm long PCL conduit (Lewis male rats)	10 mm long or 20 mm long Mg^2+^ filament was inserted inside conduits, extending two mm into both stumps
Li et al., 2016 [164]	Acute sciatic nerve compression performed using an 8 mm long silastic tube for 3 h. (Sprague–Dawley rats)	A biodegradable Mg-3%Al-1%Zn wire (diameter 3 mm) was put through the sciatic nerve epineurium
Vennemeyer et al., 2015 [165]	6 mm sciatic nerve defect repaired with 8 mm long poly(caprolactone) (PCL) nerve conduit (Lewis male rats)	Mg filaments were embedded into the nerve stumps (2 mm deep) with the conduit threaded over all. The conduit lumens were filled via syringe with a keratin hydrogel or sterile saline.
Pan et al., 2011 [158]	Sciatic nerve crush (Imprinting control region mice)	- Low-Mg diet containing <0.08 mg/g Mg - High-Mg diet containing 0.7 mg/g Mg and supplemented by MgCl_2_ 0.5 mg/mL in water for 3 weeks before experiments and 4 weeks after nerve injury
Gougoulias et al., 2004 [162]	Sciatic nerve crush at the 2nd day postnatal (Wistar Albino rats)	Subcutaneous injection of MgSO_4_7H_2_O (0.05 mL of 1 M solution/10 g body weight) daily from operation day until rats were 14 days old
Greensmith et al., 1995 [161]	Sciatic nerve crush at the day of birth (Wistar Albino rats)	Subcutaneous injection of MgSO_4_ (0.05 mL 1 M MgSO_4_/10 g body weight) daily from operation day until rats were 10 days old
**Selenium**		
Kizilay et al., 2017 [166]	Sciatic nerve crush (Wistar albino rats)	Selenium was given at a dose of 1.5 mg/kg (dissolved in sterile saline) by oral gavage at 1st, 24th, 48th, and 72nd h after surgery
**Zinc**		
Qian et al., 2020 [167]	15 mm sciatic nerve gap repaired with ZnO-loaded PCL piezoelectric nanogenerator scaffold (Sprague–Dawley male rats)	Zinc oxide nanoparticles (purity 99%)-loaded PCL piezoelectric nanogenerator scaffold fabricated using 3D injectable multilayer moulding technique
Iman et al., 2017 [168]	15 mm sciatic nerve gap repaired with 14 mm chitosan–zinc oxide nanocomposite conduit (White Wistar male rats)	Chitosan–zinc oxide nanocomposite conduit. Analysis at 4, 8, and 12 weeks after surgery
**Calcium**		
Salehi et al., 2018 [169]	Sciatic nerve crush (Wistar male rats)	Local injection of 0.50 mL of a single batch of collagen + hydroxyapatite nanoparticles hydrogel to the site of crush injury using a 16-gauge needle
